# Supplementary material for: Model-free tracking control of complex dynamical trajectories with machine learning
Source: Nat Commun. 2023 Sep 14;14:5698. doi: 10.1038/s41467-023-41379-3 (PMC10502079; doi:10.1038/s41467-023-41379-3)
Supplement: Supplementary file 1 — Supplementary Information [file 41467_2023_41379_MOESM1_ESM.pdf]

Supplementary Information for

Model-free tracking control of complex dynamical trajectories with machine learning

Zheng-Meng Zhai, Mohammadamin Moradi, Ling-Wei Kong, Bryan Glaz, Mulugeta Haile, and Ying-Cheng Lai

Corresponding author: Ying-Cheng Lai (Ying-Cheng.Lai@asu.edu)

CONTENTS

Supplementary Note 1: Reservoir computing 2

Supplementary Note 2: Generation of a variety of reference trajectories 3
Chaotic Lorenz reference trajectory 4
Circular reference trajectory 4
Chaotic Mackey-Glass reference trajectory 4
Periodic figure-8 reference trajectory 5
Chaotic Rössler reference trajectory 5
Fermat's spiral reference trajectory 5
Heart-shaped reference trajectory 5
Astroid reference trajectory 6
Two types of chaotic trajectories from dynamical systems with a polynomial velocity field 6
Epitrochoid reference trajectory 6
Chaotic Chua-circuit reference trajectory 7
Talbot reference trajectory 7
Lissajous reference trajectory 7
Chaotic Lorenz-96 reference trajectory 8

Supplementary Note 3: Bridge to reference trajectory 8

Supplementary Note 4: Alternating and continuous tracking of a variety of reference trajectories 10
Tracking Control 10
Robustness against uncertainties 11
Effect of varying training parameters 12

Supplementary Note 5: Pertinent issues of tracking control 14
Safe region of initial conditions for control success 14
Tracking speed tolerance 16
Robustness against variations in training parameters 18

Supplementary Note 6: Tracking control with feed-forward neural networks 19

Supplementary References 21

SUPPLEMENTARY NOTE 1: RESERVOIR COMPUTING

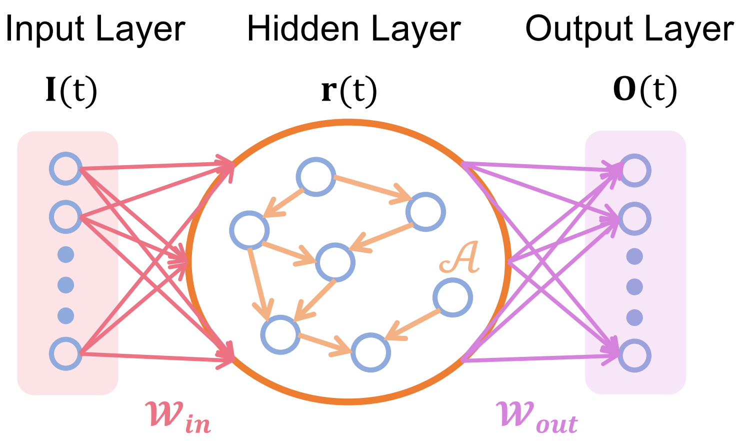

FIG. S1. Structure of reservoir computing.

The key requirement for selecting a machine-learning scheme for our tracking-control problem is to generate a control signal from a desired trajectory. When the trajectory is chaotic, the control signal can be quite complex. Reservoir computing [1–3], a type of recurrent neural network, is a suitable machine-learning approach for generating complex desired signals as output from complex input signals. Hence, it is an appropriate choice for our problem. A reservoir computer is composed of three layers: an input layer, a hidden recurrent layer, and an output layer, as shown in Fig. S1. The distinguishing feature of reservoir computing (RC) is that only the readout weights ($\mathcal{W}_{\rm out}$) are trained using a simple learning algorithm, e.g., linear regression, while the input weights ($\mathcal{W}_{\rm in}$) and recurrent connection weights within the reservoir ($\mathcal{A}$) are left untrained. This approach enables a significant reduction in computational costs for learning compared to conventional RNNs, which is the major advantage of RC. Since no backpropagation is required, the difficulty of exploding/vanishing gradients associated with the training of recurrent neural networks does not occur. As shown in Fig. S1, the matrix $\mathcal{W}_{\rm in}$ maps the reservoir input signal $\mathbf{I}(t)$ into a high-dimensional hidden layer. The reservoir network adjacency matrix $\mathcal{A}$ usually satisfies "the echo-state property" where the dynamical state of the network is confined to a single attractor [1]. The echo-state property is referred to as the ability of the reservoir to retain past information from the input signal for a limited time. This property is essential to effectively process time-varying signals. Activated by the sequence of reservoir input signals $[\mathbf{I}(1), \mathbf{I}(2), \cdots, \mathbf{I}(t)]$, the hidden layer state is updated step-by-step at the input signal time interval according to

$$\mathbf{r}(t+1) = (1-\alpha)\cdot\mathbf{r}(t) + \alpha\cdot\tanh\left[\mathcal{A}\cdot\mathbf{r}(t) + \mathcal{W}_{\rm in}\cdot\mathbf{I}(t) + \mathcal{W}_{\rm bias}\right], \tag{S1}$$

where α is the leakage parameter that determines the rate of "leakage" or "forgetting" in reservoir state updating, and the activation function is the hyperbolic tangent function ($\tanh$). The bias vector $\mathcal{W}_{\rm bias}$ is composed of equal constants $w_{\rm b}$, whose role inside the $\tanh$ function is to shift the small signals into the linear region in the activation function [4]. It is worth mentioning that a smaller leakage rate α enables the system to retain more information about past inputs, whereas a larger one makes the system more biased towards recent inputs. Let $D_{\rm i}$, $D_{\rm r}$ and $D_{\rm o}$ be the dimensions of the input signal $\mathbf{I}(t)$, the hidden network state $\mathbf{r}(t)$, and the output signal $\mathbf{O}(t)$,

respectively. The dimension of the input matrix $\mathcal{W}_{\rm in}$ is $D_{\rm r} * D_{\rm i}$, whose elements are chosen uniformly from the interval $[-\gamma, \gamma]$ prior to training. The dimension of the network adjacency matrix $\mathcal{A}$ in the hidden layer is $D_{\rm r} * D_{\rm r}$, which results in a high-dimensional, symmetric, sparse, random network with link probability p and spectral radius ρ. Typically, $D_{\rm r}$ needs to be much larger than the input dimension $D_{\rm i}$ to ensure that the network has a sufficient degree of complexity to "learn" and process complex input signals. The nonzero elements in $\mathcal{A}$ are generated by Gaussian distribution with zero mean and unit variance before training. The dimension of the output matrix $\mathcal{W}_{\rm o}$ is $D_{\rm o} * D_{\rm r}$.

To facilitate training, we calculate, update, and concatenate the vector $\mathbf{r}(t)$ characterizing the dynamical state of the reservoir network into a matrix $\mathcal{R}$ of the dimension $D_{\rm r} * T_{\rm train}$, where $T_{\rm train}$ is the training length that includes all the episodes of the training phase. The input signal is concatenated into a matrix $\mathcal{U}$. The output matrix is determined by using Tikhonov regularization [5] as

$$\mathcal{W}_{\rm out} = \mathcal{U} \cdot \mathcal{R}'^{\intercal} (\mathcal{R}' \cdot \mathcal{R}'^{\intercal} + \beta \mathcal{I})^{-1}, \tag{S2}$$

where $\mathcal{I}$ is the identity matrix of dimension $D_{\rm r}$, β is the regularization coefficient, and $\mathcal{R}'$ is the transpose of $\mathcal{R}$. In the testing phase, to measure the machine's performance and tracking accuracy, we use the difference between the current Cartesian position $[C_x, C_y]$ and the reference position $[D_x, D_y]$ as the root mean square error (RMSE):

$$\mathrm{RMSE} = \sqrt{\frac{1}{2T_{\rm test}} \sum_{t=1}^{T_{\rm test}} [(C_x(t) - D_x(t))^2 + (C_y(t) - D_y(t))^2]}, \tag{S3}$$

where $T_{\rm test}$ is the test length. The output of the reservoir neural network is

$$\mathbf{O}(t) = \mathcal{W}_{\rm out} \mathbf{r}(t). \tag{S4}$$

There are six hyperparameters to be optimized: the spectral radius of the reservoir ρ, the scaling factor of the input weights of the input matrix γ, the leakage parameter α, the regularization coefficient β, the link probability p of the reservoir network and the bias constant $w_{\rm b}$ of the bias matrix. These hyperparameters are selected by the surrogate optimization (surrogateopt) algorithm in Matlab [6].

SUPPLEMENTARY NOTE 2: GENERATION OF A VARIETY OF REFERENCE TRAJECTORIES

We describe the 15 reference trajectories (eight periodic and seven chaotic) tested in our work. All the chaotic trajectories are from three-dimensional dynamical systems. For the planar tracking problem, we use two variables.

Chaotic Lorenz reference trajectory

The classic Lorenz chaotic system [7], a simplified model for atmospheric convection, is

$$\begin{aligned}\frac{dx_{\rm lo}}{dt} &= \sigma_{\rm lo}(y_{\rm lo} - x_{\rm lo}),\\ \frac{dy_{\rm lo}}{dt} &= x_{\rm lo}(\rho_{\rm lo} - z_{\rm lo}) - y_{\rm lo},\\ \frac{dz_{\rm lo}}{dt} &= x_{\rm lo}y_{\rm lo} - \beta_{\rm lo}z_{\rm lo},\end{aligned} \tag{S5}$$

where $x_{\rm lo}$, $y_{\rm lo}$ and $z_{\rm lo}$ are proportional to the rate of convection, the horizontal and vertical temperature variation, respectively, and $\sigma_{\rm lo} > 0$, $\rho_{\rm lo} > 0$ and $\beta_{\rm lo} > 0$ are system parameters. By adjusting the three parameters, the Lorenz system can generate periodic or chaotic trajectories. We choose the parameter values as $\sigma_{\rm lo} = 10$, $\rho_{\rm lo} = 8/3$ and $\beta_{\rm lo} = 26$ to generate a chaotic reference trajectory and use $x_{\rm lo}$ and $y_{\rm lo}$ to represent a planar trajectory. We scale $x_{\rm lo}$ and $y_{\rm lo}$ to within the range $[-0.5, 0.5]$ according to

$$x_{\rm scaled} = a_{\rm s} + (b_{\rm s} - a_{\rm s}) \cdot \frac{x_o - \min(x_o)}{\max(x_o) - \min(x_o)}, \tag{S6}$$

where x_o denotes the original data, and the rescaled data is in the interval $[a_{\rm s}, b_{\rm s}]$.

Circular reference trajectory

A circular reference trajectory is periodic, which can be described as

$$\begin{aligned}x_{\rm ci} &= a_{\rm ci} \cdot \cos(\frac{2\pi t}{T_{\rm p}}),\\ y_{\rm ci} &= a_{\rm ci} \cdot \sin(\frac{2\pi t}{T_{\rm p}}),\end{aligned} \tag{S7}$$

where $a_{\rm ci}$ limits the size of the trajectory and is set to be $a_{\rm ci} = 0.5$, t is the time and $T_{\rm p} = 150$ is a parameter determining the period of the trajectory.

Chaotic Mackey-Glass reference trajectory

The Mackey-Glass system [8, 9] is an infinite dimensional system described by a delayed differential equation:

$$\dot{x}_{\rm mg}(t) = \frac{a_{\rm mg}x_{\rm mg}(t - \tau_{\rm mg})}{1 + (x_{\rm mg}(t - \tau_{\rm mg})^{c_{\rm mg}}} - b_{\rm mg}x_{\rm mg}(t), \tag{S8}$$

where $\tau_{\rm mg}$ is the delayed time, $a_{\rm mg}$, $b_{\rm mg}$ and $c_{\rm mg}$ are parameters. We set $a_{\rm mg} = 0.2$, $b_{\rm mg} = 0.1$ and $c_{\rm mg} = 10$ and choose two delayed times: $\tau_{\rm mg} = 17$ and $\tau_{\rm mg} = 30$. The system generates a low-dimensional chaotic attractor with one positive Lyapunov exponent for $\tau_{\rm mg} = 17$ and a high-dimensional chaotic attractor with two positive Lyapunov exponents for $\tau_{\rm mg} = 30$. We use Eq. (S6) to scale the data into the ranges $[-0.5, 0.5]$ and $[-0.4, 0.4]$ for $\tau_{\rm mg} = 17$ and $\tau_{\rm mg} = 30$, respectively. We use the variables $x_{\rm mg}(t)$ and $x_{\rm mg}(t-\tau_{\rm mg})$ to generate a planar reference trajectory.

Periodic figure-8 reference trajectory

The figure of eight is a periodic trajectory described by

$$x_{\mathrm{ei}} = a_{\mathrm{ei}} \cdot \sin(\frac{2\pi t}{T_{\mathrm{p}}}),$$
$$y_{\mathrm{ei}} = b_{\mathrm{ei}} \cdot \cos(\frac{2\pi t}{T_{\mathrm{p}}/2}), \tag{S9}$$

where a_{ei} and b_{ei} are the scaling factors to control the size of the figure and T_{p} is the parameter determining the period of the trajectory. We set $a_{\mathrm{ei}} = 0.25$, $b_{\mathrm{ei}} = 0.15$ and $T_{\mathrm{p}} = 150$.

Chaotic Rössler reference trajectory

The Rössler system [10] is given by

$$\frac{dx_{\mathrm{ro}}}{dt} = -y_{\mathrm{ro}} - z_{\mathrm{ro}},$$
$$\frac{dy_{\mathrm{ro}}}{dt} = x_{\mathrm{ro}} + a_{\mathrm{ro}} y_{\mathrm{ro}}, \tag{S10}$$
$$\frac{dz_{\mathrm{ro}}}{dt} = b_{\mathrm{ro}} + z_{\mathrm{ro}}(x_{\mathrm{ro}} - c_{\mathrm{ro}}),$$

where a_{ro}, b_{ro} and c_{ro} are system parameters. For $a_{\mathrm{ro}} = 0.2$, $b_{\mathrm{ro}} = 0.2$ and $c_{\mathrm{ro}} = 5.7$, the system generates a chaotic attractor. We take x_{ro} and b_{ro}, normalized into the range $[-0.35, 0.35]$ according to Eq. S6, as the dynamical variables associated with the chaotic reference trajectory.

Fermat's spiral reference trajectory

Fermat's spiral [11] has the property that the area between two consecutive full turns around the spiral is invariant. It is described in the polar coordinates as

$$r_{\mathrm{fs}}^2 = a_{\mathrm{fs}}^2 \theta_{\mathrm{fs}}, \tag{S11}$$

where $(r_{\mathrm{fs}}, \theta_{\mathrm{fs}})$ are the polar coordinates and a_{fs} is a scaling factor. We set $a_{\mathrm{fs}} = 0.5$ and $\theta_{\mathrm{fs}} = 2\pi t / f_{\mathrm{fs}}$, where $f_{\mathrm{fs}} = 100$ is set to adjust the speed of the trajectory. In the Cartesian coordinates, the trajectory is given by

$$x = r \cdot \cos(\theta),$$
$$y = r \cdot \sin(\theta), \tag{S12}$$

Heart-shaped reference trajectory

A heart-shaped curve can be described in the polar coordinates as

$$r_{\mathrm{he}}(\theta_{\mathrm{he}}) = 1 - \sin(\theta_{\mathrm{he}}). \tag{S13}$$

We set $\theta_{\mathrm{he}} = 2\pi t / f_{\mathrm{he}}$, where $f_{\mathrm{he}} = 250$ is used to adjust the speed of the trajectory. In the Cartesian coordinates, the trajectory is described by Eq. (S12).

Astroid reference trajectory

In mathematics, an astroid is a type of roulette curves and a hypocycloid with four cusps. The curve can be described parametrically as

$$\begin{aligned} x_{\mathrm{as}} &= a_{\mathrm{as}} \cos^3(\theta_{\mathrm{as}}), \\ y_{\mathrm{as}} &= a_{\mathrm{as}} \sin^3(\theta_{\mathrm{as}}), \end{aligned} \tag{S14}$$

where a_{as} is a scaling factor. we choose $a_{\mathrm{as}} = 0.4$ and $\theta_{\mathrm{as}} = 2\pi t/f_{\mathrm{as}}$, where $f_{\mathrm{as}} = 250$ is used to adjust the speed of the trajectory.

Two types of chaotic trajectories from dynamical systems with a polynomial velocity field

In Ref. [12], 19 distinct examples of chaotic attractors are listed. We choose two examples with the corresponding differential equations given by

$$\begin{aligned} \frac{dx_{\mathrm{sp}}}{dt} &= y_{\mathrm{sp}}, \\ \frac{dy_{\mathrm{sp}}}{dt} &= -x_{\mathrm{sp}} + y_{\mathrm{sp}} z_{\mathrm{sp}}, \\ \frac{dz_{\mathrm{sp}}}{dt} &= 1 - y_{\mathrm{sp}}^2, \end{aligned} \tag{S15}$$

and

$$\begin{aligned} \frac{dx_{\mathrm{sp}}}{dt} &= -y_{\mathrm{sp}}, \\ \frac{dy_{\mathrm{sp}}}{dt} &= x_{\mathrm{sp}} - y_{\mathrm{sp}}, \\ \frac{dz_{\mathrm{sp}}}{dt} &= x_{\mathrm{sp}} z_{\mathrm{sp}} + 3y_{\mathrm{sp}}^2. \end{aligned} \tag{S16}$$

We take the x_{sp} and b_{sp} as the dynamical variables for the chaotic reference trajectories in the plane and scale them into the range $[-0.4, 0.4]$ according to Eq. S6.

Epitrochoid reference trajectory

Epitrochoid is a kind of roulette curve generated by tracing the center of the circle with radius r which rolls around the outside of a fixed circle of radius R. Parametrically, an epitrochoid can be described as

$$\begin{aligned} x_{\mathrm{ep}} &= (a_{\mathrm{ep}} + b_{\mathrm{ep}}) \cos(\theta_{\mathrm{ep}}) - c_{\mathrm{ep}} \cos((\frac{a_{\mathrm{ep}}}{b_{\mathrm{ep}}} + 1)\theta_{\mathrm{ep}}), \\ y_{\mathrm{ep}} &= (a_{\mathrm{ep}} + b_{\mathrm{ep}}) \sin(\theta_{\mathrm{ep}}) - c_{\mathrm{ep}} \sin((\frac{a_{\mathrm{ep}}}{b_{\mathrm{ep}}} + 1)\theta_{\mathrm{ep}}), \end{aligned} \tag{S17}$$

where a_{ep}, b_{ep} and c_{ep} are parameters. We choose $a_{\mathrm{ep}} = 5$, $b_{\mathrm{ep}} = 3$, $c_{\mathrm{ep}} = 5$, and $\theta_{\mathrm{ep}} = 2\pi t/f_{\mathrm{as}}$ with $f_{\mathrm{as}} = 200$ to tune the speed of the trajectory.

Chaotic Chua-circuit reference trajectory

Chua's circuit can generate a double-scroll chaotic attractor [13]. One set of differential equations describing the Chua's circuit is

$$\begin{aligned}
\frac{dx_{\text{ch}}}{dt} &= \alpha_{\text{ch}}(y_{\text{ch}} - x_{\text{ch}} - h_{\text{ch}}), \\
\frac{dy_{\text{ch}}}{dt} &= x_{\text{ch}} - y_{\text{ch}} + z_{\text{ch}}, \\
\frac{dz_{\text{ch}}}{dt} &= -\beta_{\text{ch}} y_{\text{ch}},
\end{aligned} \tag{S18}$$

where

$$h_{\text{ch}} = \mu_{\text{ch}_1} x_{\text{ch}} + 0.5(\mu_{\text{ch}_0} - \mu_{\text{ch}_1}) \cdot (|x_{\text{ch}} + 1| - |x_{\text{ch}} - 1|),$$

α_{ch}, β_{ch}, μ_{ch_0} and μ_{ch_1} are parameters. We choose $\alpha_{\text{ch}} = 14.405$, $\beta_{\text{ch}} = 28$, $\mu_{\text{ch}_0} = -1.143$ and $\mu_{\text{ch}_1} = -0.714$ to generate a chaotic attractor, with the variables x_{ch} and y_{ch} scaled into the respective ranges $[-0.5, 0.5]$ and $[-0.3, 0.3]$ representing the reference trajectory.

Talbot reference trajectory

The Talbot curve is a negative pedal of an ellipse with respect to the ellipse's center [14]. Parametrically, the curve is described as

$$\begin{aligned}
x_{\text{ta}} &= \frac{(a_{\text{ta}}^2 + c_{\text{ta}}^2 \sin^2(\theta_{\text{ta}})) \cos(\theta_{\text{ta}})}{a_{\text{ta}}}, \\
y_{\text{ta}} &= \frac{(a_{\text{ta}}^2 - 2c_{\text{ta}}^2 + c_{\text{ta}}^2 \sin^2(\theta_{\text{ta}})) \sin(t_{\text{ta}})}{b_{\text{ta}}},
\end{aligned} \tag{S19}$$

where a_{ta}, b_{ta} and c_{ta} are parameters. We choose $a_{\text{ta}} = 1.1$, $b_{\text{ta}} = 0.666$, $c_{\text{ta}} = 1$, and $\theta_{\text{ta}} = 2\pi t / f_{\text{as}}$ with $f_{\text{as}} = 400$ to set the speed of the curve. The coordinates x_{ta} and y_{ta} are scaled into the respective ranges $[-0.4, 0.4]$ and $[-0.3, 0.3]$.

Lissajous reference trajectory

A Lissajous curve, also called a Lissajous figure or Bowditch curve, is described by the following parametric equations

$$\begin{aligned}
x_{\text{li}} &= A_{\text{li}} \sin(a_{\text{li}} \theta_{\text{li}} + c_{\text{li}}), \\
y_{\text{li}} &= B_{\text{li}} \sin(b_{\text{li}} \theta_{\text{li}}),
\end{aligned} \tag{S20}$$

where A_{li}, B_{li}, a_{li}, b_{li} and c_{li} are parameters that control the shape of the Lissajous curve. We choose $A_{\text{li}} = 1$, $B_{\text{li}} = 1$, $a_{\text{li}} = 1$, $b_{\text{li}} = 3$, $c_{\text{li}} = \pi/4$, and $\theta_{\text{li}} = 2\pi t / f_{\text{li}}$ with $f_{\text{li}} = 300$ to set the speed of the trajectory. The coordinates x_{li} and y_{li} are scaled into the range $[-0.3, 0.3]$.

Chaotic Lorenz-96 reference trajectory

The Lorenz-96 model [15] is a widely used benchmark dynamical system in meteorological and climatology research. It provides an abstract but effective representation of the atmospheric dynamics and exhibits complex behaviors similar to that observed in real atmospheric phenomena. The high-dimensional state vector is $X_{196} = [x_{196}^0, \cdots, x_{196}^{N-1}]^T$ with the governing equation [16]

$$\frac{dx_{196}^n}{dt} = (x_{196}^{n+1} - x_{196}^{n-2})x_{196}^{n-1} - x_{196}^n + F \tag{S21}$$

for $n \in \{0, 1, \cdots, N-1\}$, where the boundary conditions are $x_{196}^{-1} = x_{196}^{N-1}, x_{196}^{-2} = x_{196}^{N-2}$ and F is the forcing amplitude. We set $N = 40$ and $F = 8$. Since the two-arm manipulator is in a 2D plane, we choose the projection of the trajectory in the first two dimensions as the reference trajectory scaled into the respective ranges [-0.6, 0.6] and [-0.7, 0.7].

SUPPLEMENTARY NOTE 3: BRIDGE TO REFERENCE TRAJECTORY

It is often the case that the starting position of the end effector is not on the given reference trajectory, requiring a "bridge" to drive the end effector from the starting position to the trajectory. Let T_x and T_y be the closest points of the reference trajectory to the starting position C_x, C_y of the end effector. The required time to "cross the bridge" is $t_b = \sqrt{(C_x - T_x)^2 + (C_y - T_y)^2}/dt$. Since a smooth trajectory in the Cartesian coordinates may not lead to a smooth trajectory in the polar coordinates, we convert the two points into angular positions according to Eqs. (7) and (8) in the main text. This way, the bridge will be in the angular space, i.e., we generate a series of points traveling from the initial point to the reference trajectory in terms of the angles.

A method to build the bridge is through cubic polynomial trajectory generation [17]. In particular, consider the cubic polynomial of the form

$$q(t) = a_0 + a_1 t + a_2 t^2 + a_3 t^3, \tag{S22}$$

where $q(t) = [q_1(t), q_2(t)]$ is the angular position. Setting the velocities of the initial and the end point as $\dot{q}(0)$ and $\dot{q}(t_b)$, we can write the equations to describe the cubic polynomial as

$$\begin{aligned} q(0) &= a_0, \\ q(t_b) &= a_0 + a_1 t_b + a_2 t_b^2 + a_3 t_b^3, \\ \dot{q}(0) &= a_1, \\ \dot{q}(t_b) &= a_1 + 2a_2 t_b + 3a_3 t_b^2. \end{aligned} \tag{S23}$$

The solutions are

$$\begin{aligned} a_0 &= q(0), \\ a_1 &= \dot{q}(0), \\ a_2 &= \frac{3}{t_b^2}(q(t_b) - q(0)) - \frac{2}{t_b}\dot{q}(0) - \frac{1}{t_b}\dot{q}(t_b), \\ a_3 &= -\frac{2}{t_b^3}(q(t_b) - q(0)) + \frac{1}{t_b^2}(\dot{q}(t_b) + \dot{q}(0)). \end{aligned} \tag{S24}$$

Given the angular positions and velocities of both robot arms, we can then build a smooth "bridge" in the angular space using Eq. (S24) to make the robot arm move smoothly and gradually approach the starting section of the trajectory.

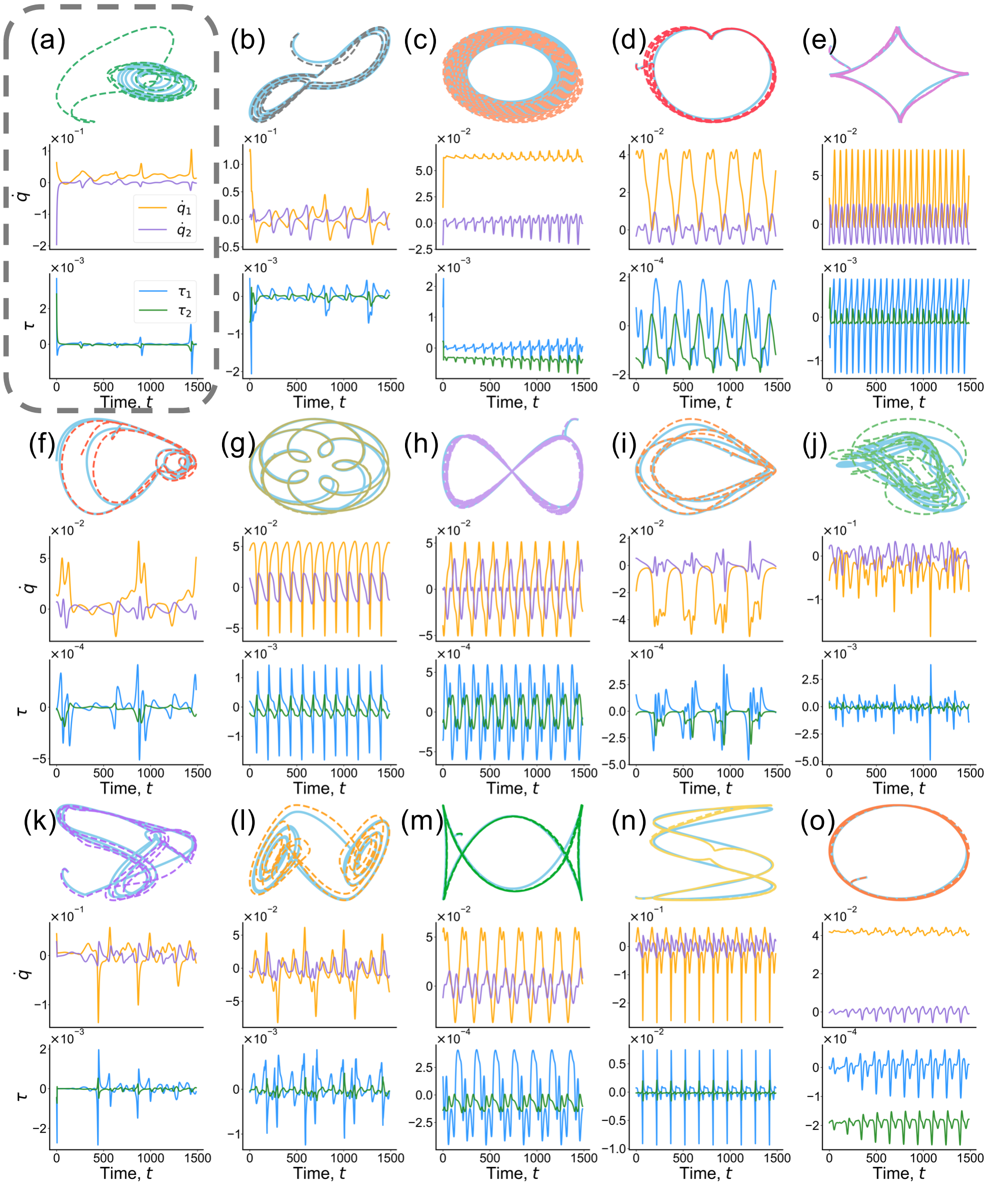

FIG. S2. Tracking control of 15 different kinds of reference trajectories: (a) chaotic Rössler, (b) chaotic Lorenz, (c) Fermat's spiral, (d) heart, (e) astroid, (f) polynomial chaotic, (g) epitrochoid, (h) eight symbol, (i) a different type of polynomial chaotic, (j) chaotic Mackey-Glass with $\tau_{\rm mg} = 17$, (k) chaotic Mackey-Glass with $\tau_{\rm mg} = 30$, (l) chaotic Chua, (m) Talbot, (n) Lissajous, and (o) circle. For each reference trajectory, Tracking of the position (C_x, C_y), velocity $(\dot{q})$, and the control signal (τ) from the reservoir controller are shown. The reference and tracked trajectories are represented by solid and dotted traces, respectively.

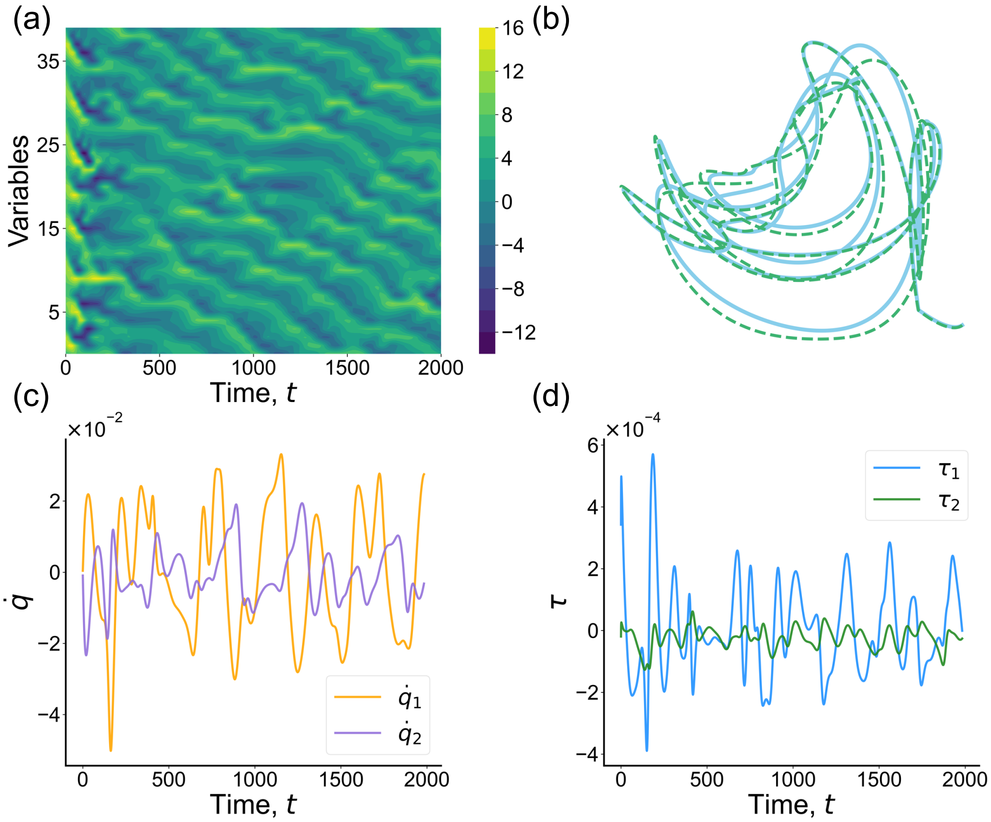

FIG. S3. Tracking control of Lorenz-96 reference trajectories. (a) spatiotemporal evolution of the chaotic Lorenz-96 system, (b) tracking of the position of the Lorenz-96 trajectory in the plane of the first and second variables, where the reference and tracked trajectories are represented by solid and dotted traces, respectively. (c,d) tracking of the speed variable ($\dot{q}$) and the corresponding control signal (τ) from the reservoir controller.

SUPPLEMENTARY NOTE 4: ALTERNATING AND CONTINUOUS TRACKING OF A VARIETY OF REFERENCE TRAJECTORIES

We present results of continuously tracking 16 different types of regular or chaotic reference trajectories, one after another, and address pertinent issues such as robustness, tracking speed tolerance, and variations in the training parameters.

Tracking Control

Figure S2 shows the results of continuous tracking of 15 different types of reference trajectories, regular or chaotic. For the first trajectory, we set all initial states including that of the reservoir controller to zero. For the remaining 14 reference trajectories, the initial states are the end states associated with the preceding trajectory. For each trajectory, we build a bridge to guide the robot arms to move forward to the trajectory. These results demonstrate the power and great flexibility of our reservoir-computing based tracking control.

To further demonstrate the generalization ability of our controller, we study an additional high-

dimensional nonlinear chaotic system: the Lorenz-96 system. Since our two-link robot arm manipulator operates in the 2D plane, we use the first two dynamical variables of the system to define a reference trajectory. Figure S3 shows the results of tracking this chaotic Lorenz-96 reference trajectory. Again, the machine-learning controller is trained with random-walk dynamics using a stochastic input control signal. In the testing phase, the controller is able to track the given complex trajectory without any fine-tuning or modification in the parameters.

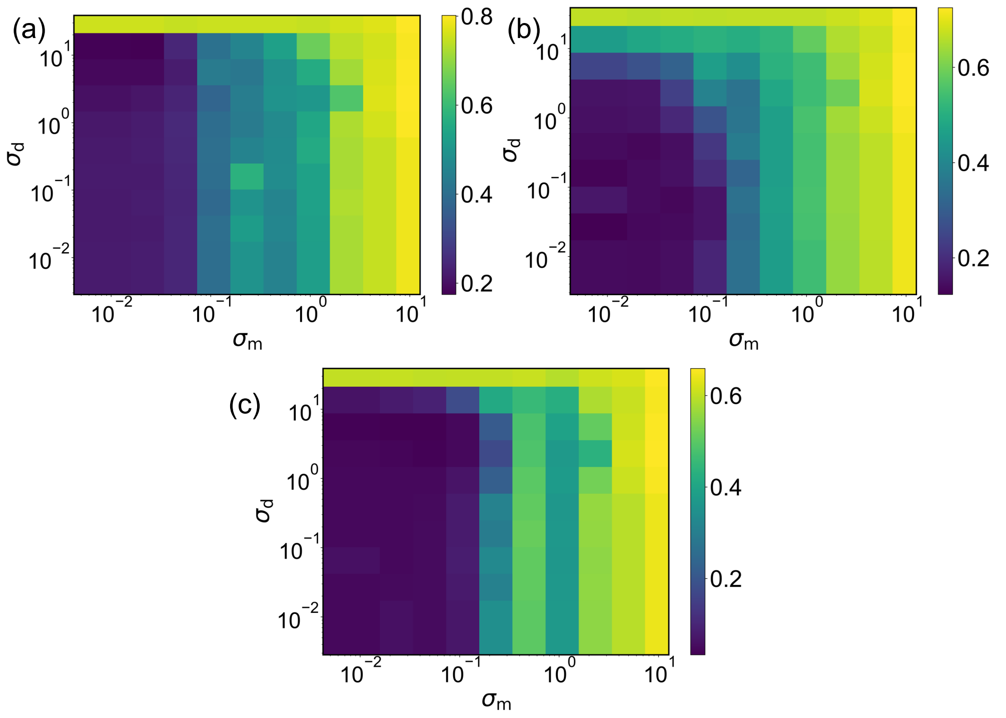

FIG. S4. Robustness against disturbance and measurement noise. Their respective amplitudes are denoted as σ_d and σ_m. (a-c) Results of robustness test with respect to the circular, chaotic Mackey-Glass with $\tau_\mathrm{mg} = 17$ and eight symbol reference trajectories, respectively. The color represents the ensemble-averaged RMSE values from 50 independent realizations. The results indicate that the reservoir-computing based tracking control framework is robust against disturbance and noise.

We present the results of robustness against disturbance and noises for three reference trajectories: circle, chaotic Mackey-Glass with $\tau_\mathrm{mg} = 17$, and the eight symbol, as shown in Fig. S4. The RMSE threshold is set to be $\mathrm{RMSE}_\mathrm{thre} = 0.18$. It can be seen that, for the three trajectories, the control system is robust against measurement noise of amplitude below 10^{-1} and disturbance of magnitude below 0.5×10^1.

Robustness against uncertainties

We further test the robustness of tracking control against parameter uncertainties. To be concrete, we assume that the uncertainty occurs in the lengths of the robot arms. Figures S5(a) and S5(b) show the results of tracking a circular and the chaotic Mackey-Glass with $\tau_\mathrm{mg} = 17$ reference trajectories, respectively. For the circular trajectory, the system is able to maintain stable

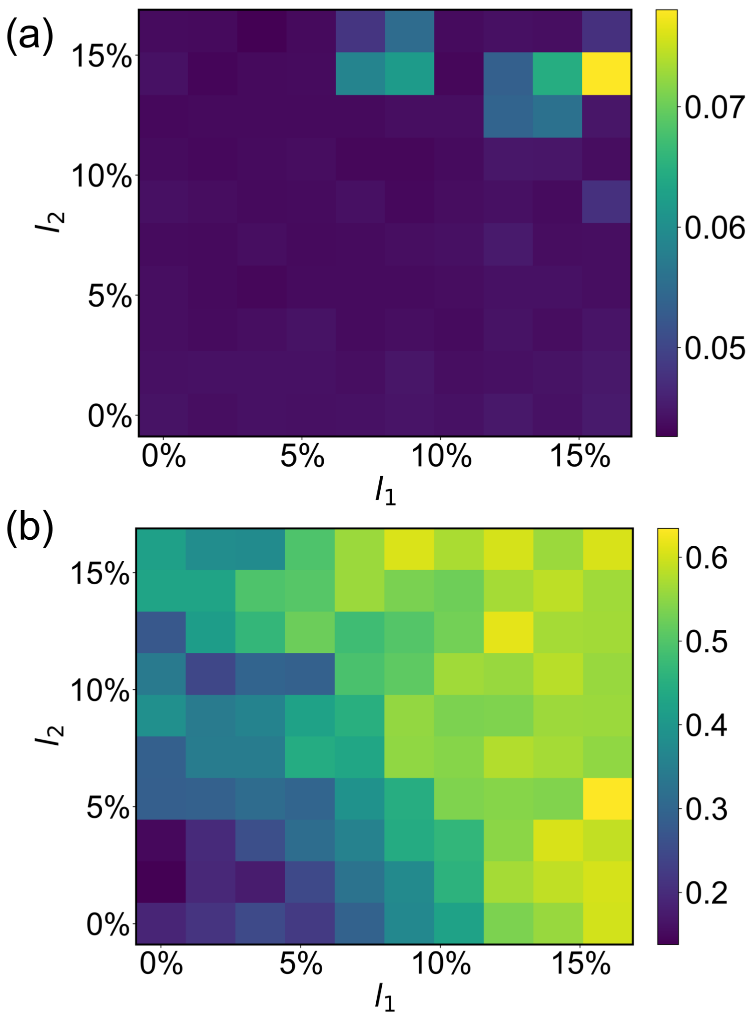

FIG. S5. Robustness against parameter uncertainty. (a,b) Tracking a circular and the chaotic Mackey-Glass with $\tau_{\mathrm{mg}} = 17$ reference trajectories, respectively. The controller is trained with the arm lengths $l_1 = 0.5$ and $l_2 = 0.5$, but tested with different values of l_1 and l_2. The color represents the ensemble averaged values of RMSE (from 50 realizations). Insofar as the reference trajectory does not come arbitrarily close to the origin, stable tracking-control performance can be achieved.

tracking in spite of the difference in the robot arm lengths. For the chaotic Mackey-Glass trajectory, the parameter uncertainty has a devastating effect on tracking control. The reason is that this trajectory can be quite to the origin, so even a small difference in the arm length will make it impossible for the end effector to come close to the origin. (For the figure-eight reference trajectory, since it crosses the origin, even a slight difference in the arm lengths l_1 and l_2 will make it impossible for the end effector to approach the origin.)

Effect of varying training parameters

We evaluate the computational time and the performance of the machine-learning based tracking controller with respect to variations in the training parameters. Figure S6(a) shows, the color-

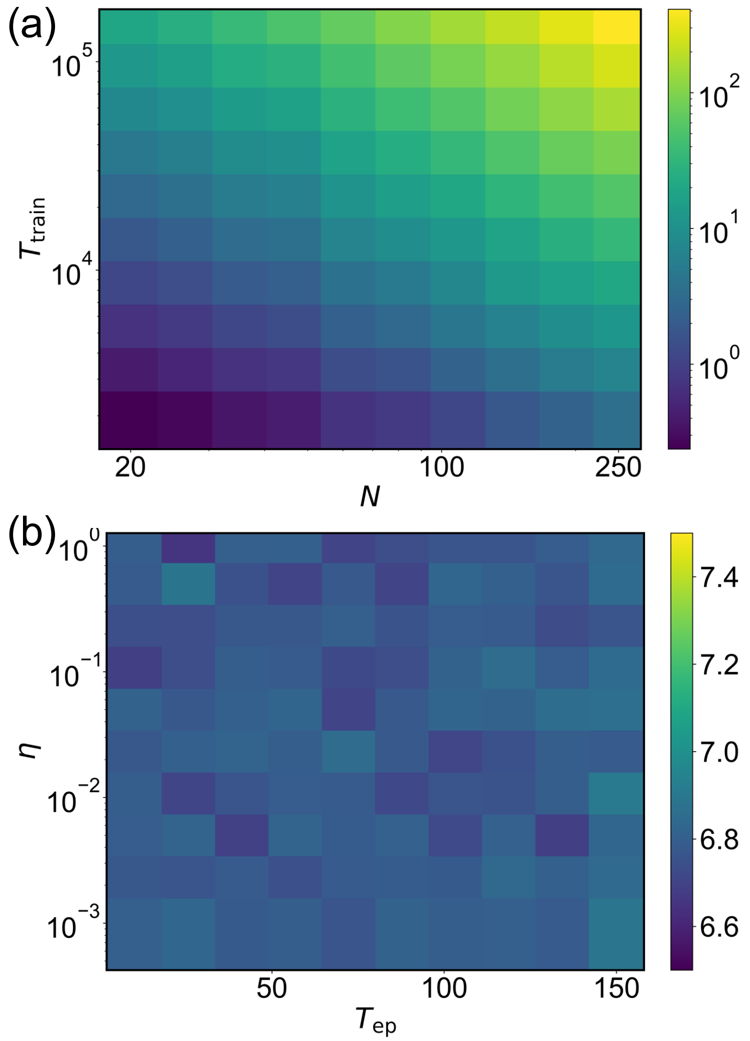

FIG. S6. Required computational time with different parameters in the training phase, averaged over the four reference trajectories in Fig. 6 in the main text. Shown is the training time required for 50 runs with variations (a) in the neural-network size N and training time T_{train}, and (b) episode length T_{ep} and the amplitude η of the stochastic, uniformly distributed control signal employed in the training. With proper choices of the parameters in machine learning and training, satisfactory training can be realized.

coded computation time required for the training phase in the parameter plane of training length T_{train} and network size N, which is averaged over the four reference trajectories in Fig. 6 in the main text. As either T_{train} or N increases, the computation time grows in an algebraic (not exponential) fashion. Figure S6(b) shows the computation time in the parameter plane (T_{ep}, η). It can be seen that there is little dependence of the time on these parameters. Figures S7(a-c) show the performance versus N and T_{train}, for the circular, chaotic Mackey-Glass with $\tau_{\mathrm{mg}} = 17$ and figure-eight reference trajectories, respectively. It can be seen that a larger network will lead to better tracking performance, while the training length T_{train}, insofar it is reasonable, has little effect on the performance. Likewise, the changes in the training episode length T_{ep} and different values of the amplitude η of the stochastic control input used in training, insofar as they are in a reasonable range, do not have a severe effect on the training performance. Figures S8(a-c) show the perfor-

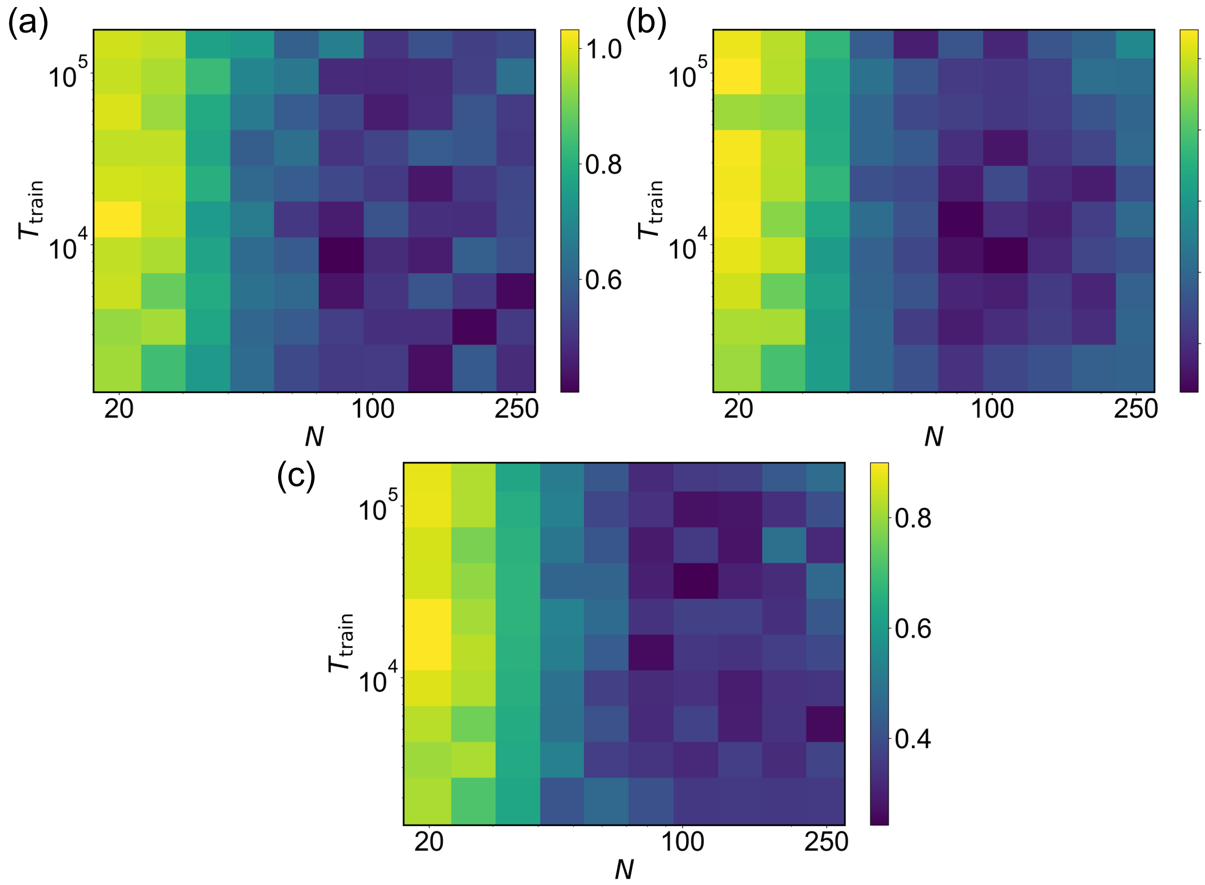

FIG. S7. Effects of network size and training time on performance. (a-c) Ensemble-averaged RMSE obtained from 50 trials versus changes in the network size N and training time T_{train} for the circular, chaotic Mackey-Glass with $\tau_{\text{mg}} = 17$ and figure-eight reference trajectories, respectively. There exist appropriate parameter ranges for satisfactory performance.

mance with respect to varying the episode length T_{ep} and the amplitude η of the stochastic control input signal employed in the training phase for the circular, chaotic Mackey-Glass system with $\tau_{\text{mg}} = 17$ and the figure-eight reference trajectories, respectively. It can be seen that appropriate combinations of T_{ep} and η should be chosen to guarantee satisfactory performance. We emphasize that our goal is not to obtain an average well performance among the 50 trails of training. On the contrary, we aim at choosing a reservoir computer trained with optimal hyperparameters and applying it in the experiment. Similar to reinforcement learning, the well-trained reservoir computer is an intelligent agent and is able to handle the different tracking tasks in practice.

SUPPLEMENTARY NOTE 5: PERTINENT ISSUES OF TRACKING CONTROL

Safe region of initial conditions for control success

For each episode of training, the initial arm positions are randomly selected. In general, the tracking success rate in the testing phase would depend on the choice of the initial arm positions. It is desired that control tracking be successful for arbitrary choices of the initial condition. Here

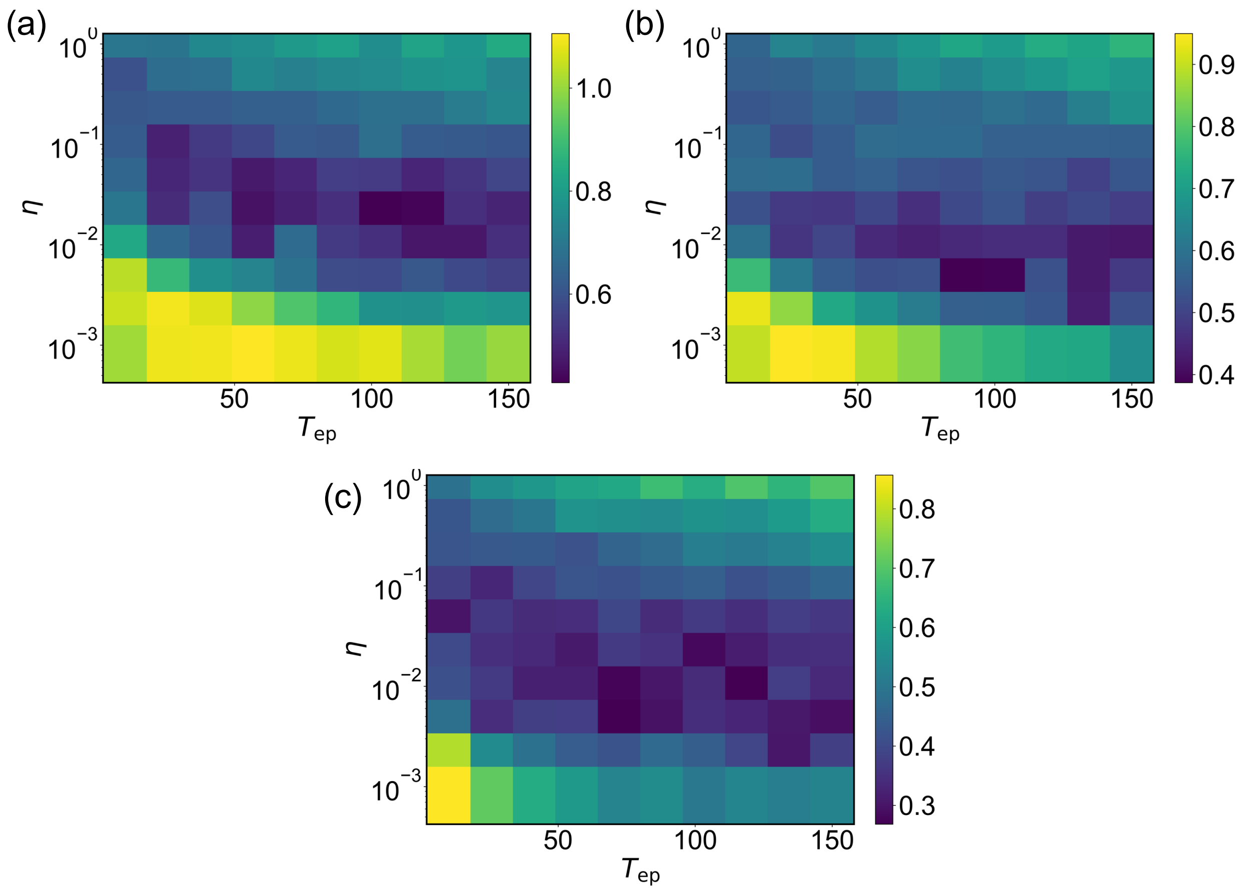

FIG. S8. Effects of episode length and stochastic input control signal on performance. (a-c) Ensemble-averaged RMSE obtained from 50 trials versus changes in the episode length T_{ep} and the amplitude η of the stochastic control input signal employed in the training phase for the circular, chaotic Mackey-Glass with $\tau_{\mathrm{mg}} = 17$ and figure-eight reference trajectories, respectively. There exist appropriate parameter ranges for satisfactory performance.

we introduce the concept of "safe region" to meet this requirement, where any initial condition from this region can guarantee a higher tracking success probability. We find that, if the end effector is initialized in the third or fourth quadrant, the success rate will increase dramatically compared with initial conditions from other regions. Figures S9(a) and S9(b) illustrate a random initial state of the two-arm system and a state from the safe region, respectively. In each case, the q_1 and q_2 coordinates are confined in the yellow and purple region with the ranges $[4, 6]$ and $[-0.1, -2.5]$, respectively, for 15 different types of reference trajectories. Figure S9(c) shows the tracking success rate of these trajectories for random (orange) and safe (blue) region initialization. For each trajectory, tracking control is carried out 100 times and the success rate is calculated as $P = N_{\mathrm{s}}/100$, where N_{s} is the number of trials in which the RMSE is smaller than a small threshold value $\mathrm{RMSE}_{\mathrm{thre}} = 0.18$. It can be seen that, if the robot arms are prepared from an initial condition in the safe region, near perfect success rate can be achieved.

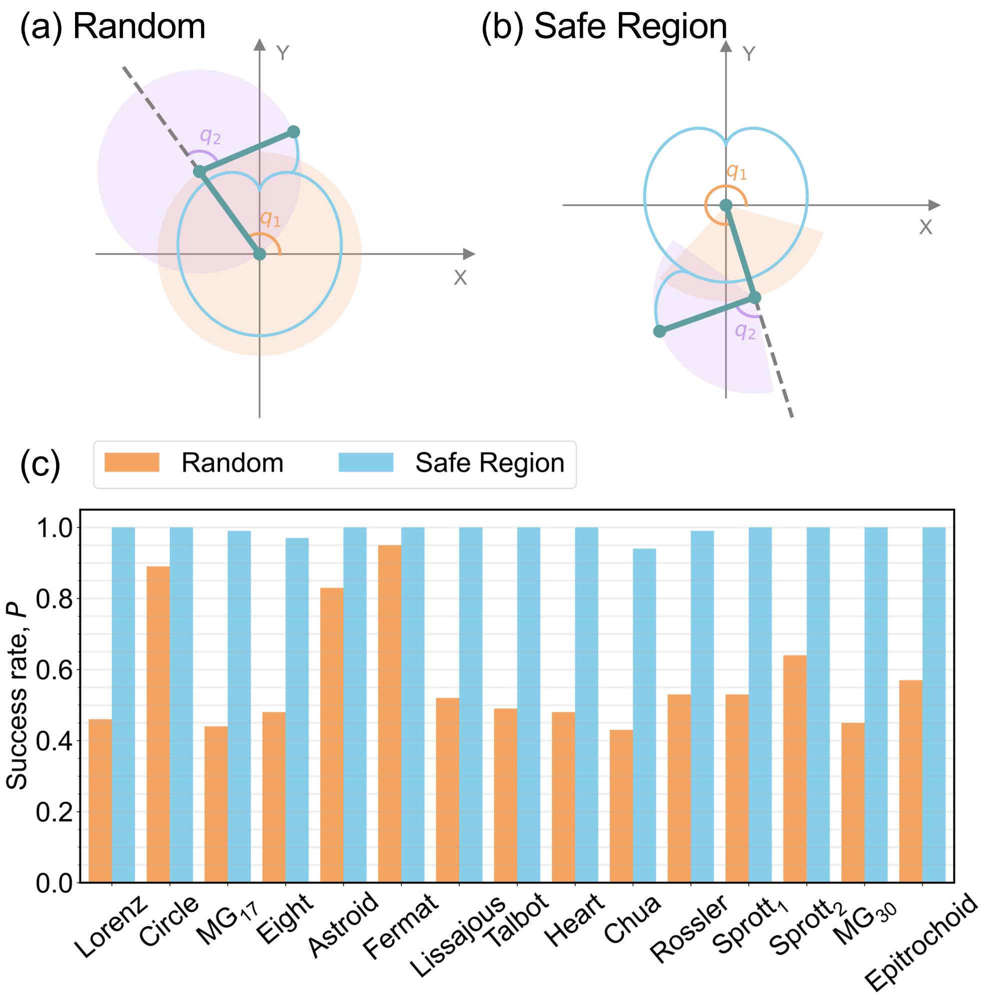

FIG. S9. Safe region for initializing the robot arms and tracking success. (a) A random initial state of the two-arm system. (b) An initial state in the safe region. (c) Tracking success rate (P) for initial conditions from the safe region (blue) compared with that for random initial conditions (orange). Preparing the dynamical system from initial conditions from the safe region for training the reservoir controller can guarantee nearly perfect success rate.

Tracking speed tolerance

During the training, the amplitude of the generated noise fed into the two-arm system as the control input varies in the range of $[-2\times10^{-2}, 2\times10^{2}]$. How fast can the noisy torques be applied? That is, what is the limit to the tracking speed? To test the tolerance of the trajectory tracking speed, we take two periodic trajectories: circle and the eight symbol with period T_{p} as shown in Figs. S10(a) and S10(b), respectively, for three different values of T_{p}. For a fixed period, we conduct tracking control 50 times, each of duration $15,000dt$. For $T_{\mathrm{p}} = 200$, tracking is accurate. However, for $T_{\mathrm{p}} = 20$ and $T_{\mathrm{p}} = 400$, significant tracking errors arise. The ensemble-averaged RMSE and the standard deviation are shown in Fig. S10(c). It can be seen that when the period is within the range $T_{\mathrm{p}} \in [100, 300]$, acceptable performance is achieved, where an acceptable case is one in which the average RMSE is below some empirical threshold, e.g., $\mathrm{RMSE}_{\mathrm{thre}} = 0.18$ (the

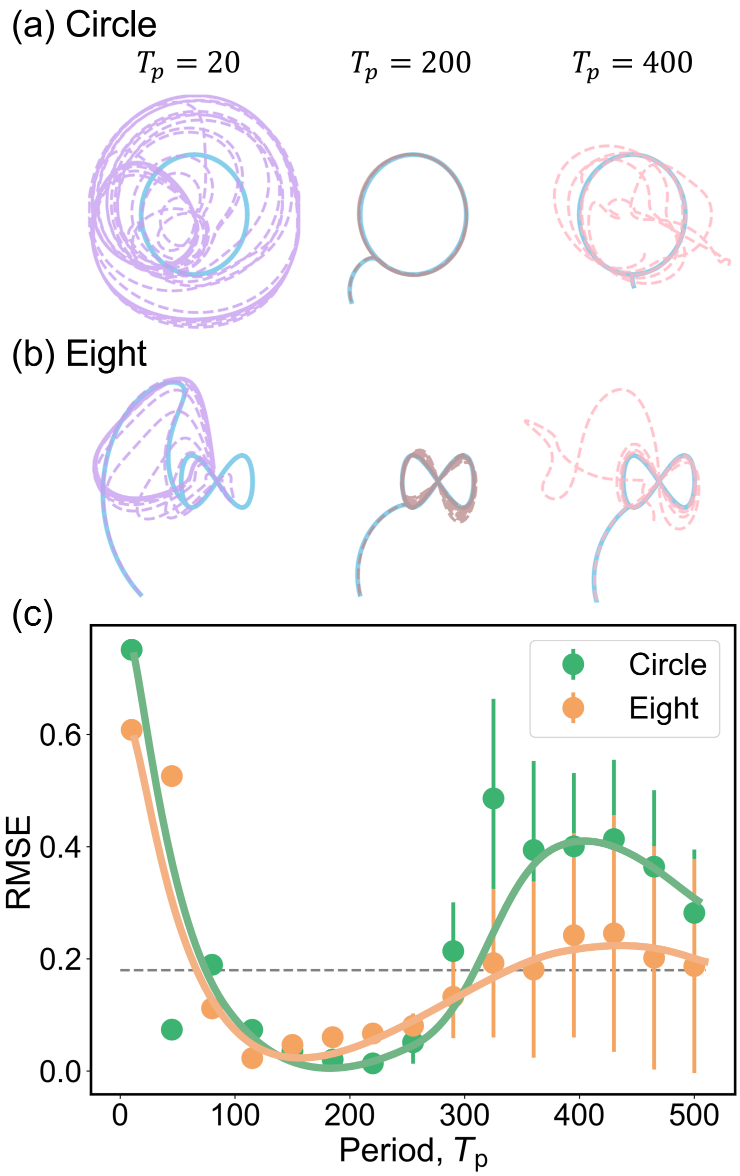

FIG. S10. Speed tolerance for successful tracking control. (a,b) Two types of periodic reference trajectories (circle and eight figure, respectively), for three different values of the period T_p. (c) Ensemble-averaged RMSE versus the period T_p for the two types of trajectories. The error bars represent the standard deviations calculated from $n = 50$ independent realizations.

horizontal dotted line). The ability to track a fast reference signal depends on the amplitude of the generated noise and the episode reset time T_ep. The reservoir machine can learn more relevant information from tracking a reference with a specific speed range. However, this necessitates a larger exploration space.

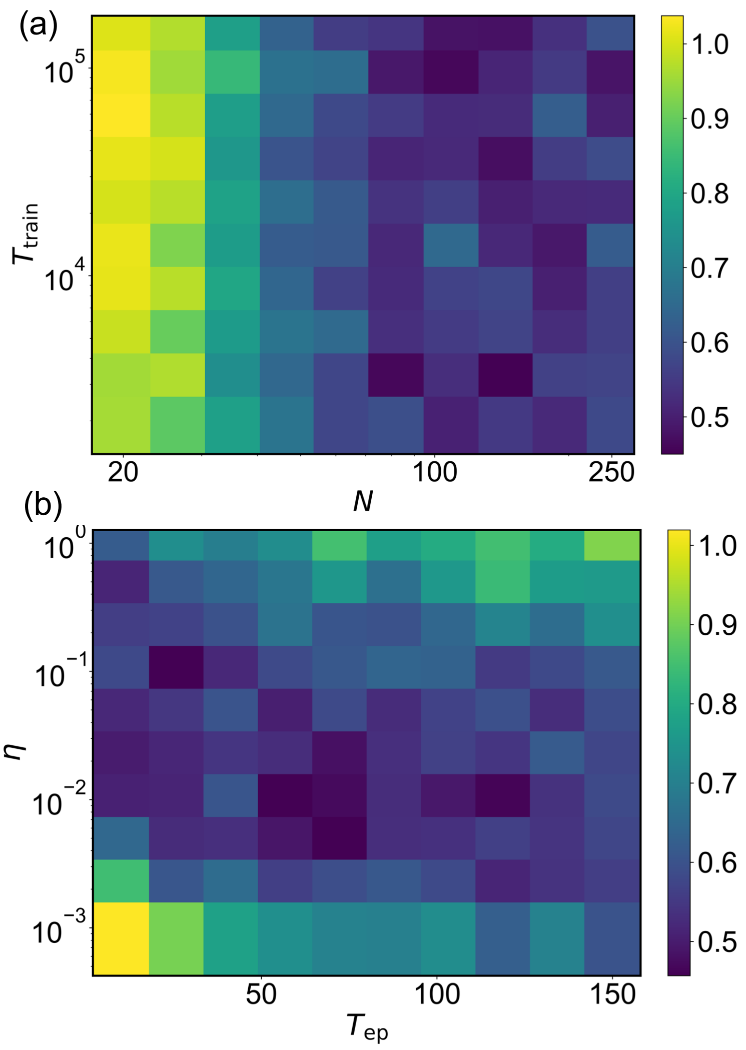

FIG. S11. Impact of variations of certain training parameters on tracking-control performance. (a) For fixed episode length $T_{\text{ep}} = 80$ and input stochastic control amplitude $\eta = 2 \times 10^{-2}$, Ensemble-averaged RMSE as the network size N and training time T_{train} vary. (b) For fixed $N = 100$ and $T_{\text{train}} = 10000$, RMSE in the parameter plane (T_{ep}, η). In both (a) and (b), each value of the RMSE is obtained from 50 independent experiments.

Robustness against variations in training parameters

A proper choice of the key parameters involved in the training is essential to the working of the reservoir controller, and it is important to assess the impact of the parameter variations on the tracking performance. Here we present results with varying four such parameters for tracking a chaotic Lorenz trajectory: reservoir controller network size N, training length T_{train}, episode length T_{ep}, and amplitude η of the stochastic input control signal. We consider two scenarios where, in each case, we fix two parameters, vary the other two systematically, and analyze the tracking performance. Figure S11(a) shows, for fixed $T_{\text{ep}} = 80$ and $\eta = 2 \times 10^{-2}$, color-coded rate of successful tracking control defined as RMSE < 0.18 in the parameter plane (N, T_{train}), where each value of the RMSE is obtained from 50 independent trials. It can be seen that the control

performance depends more on the network size N: if the network size is too small (e.g.,$\lesssim 100$, the RMSE is low. In fact, insofar as the network size is above about 100, the RMSE is high for a wide range of T_{train} values. Figure S11(b) shows, for fixed $N = 100$ and $T_{\text{train}} = 10000$, the RMSE in the plane (T_{ep}, η). The result suggests that the choice of the episode length T_{ep} and the noise amplitude η is important for the training success.

SUPPLEMENTARY NOTE 6: TRACKING CONTROL WITH FEED-FORWARD NEURAL NETWORKS

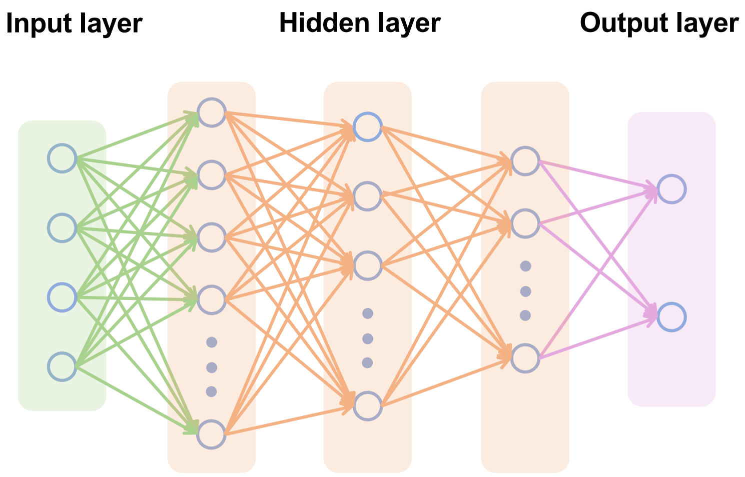

FIG. S12. Architecture of the feed-forward neural network to replace reservoir computing for tracking control. The FNN includes one input layer, three hidden layers, and one output layer.

Feed-forward neural networks (FNNs) are networks that have directed connections between artificial neurons and do not possess any loop structure [18]: information flows only in the forward direction. In artificial intelligence, FNNs have been widely used. Here we test if FNNs can replace reservoir computing for tracking control. In particular, we consider a standard FNN with a classic layered architecture: an input layer, several hidden layers, and an output layer, as shown in Fig. S12. The forward direction is the direction from the input to the output layer. Each layer consists of a number of neurons and there are only inter-layer connections, which are directed and weighted, from neurons in one layer to another in the forward direction. A sufficiently large FNN can perform as a universal approximator, which can capture the hidden complex relationship between the input and output data through a training process in which the connection weights are adjusted to achieve the desired output. We use the following hyperbolic tangent sigmoid as the activation function that compresses the output into the range $[-1, 1]$:

$$\text{tansig}(x_{\tan}) = \frac{2}{1 + e^{-2x_{\tan}}} - 1. \tag{S25}$$

We exploit FNN to construct a mapping between the error terms and the control signal required for tracking, similar to the system configuration in Fig. 4(a) in the main text. We use scaled conjugate gradient backpropagation (SCG) [19] to perform the training. In the three hidden layers, the respective number of neurons are $[50, 30, 10]$. Subtracting the observed state $\mathbf{y}(t)$ from the

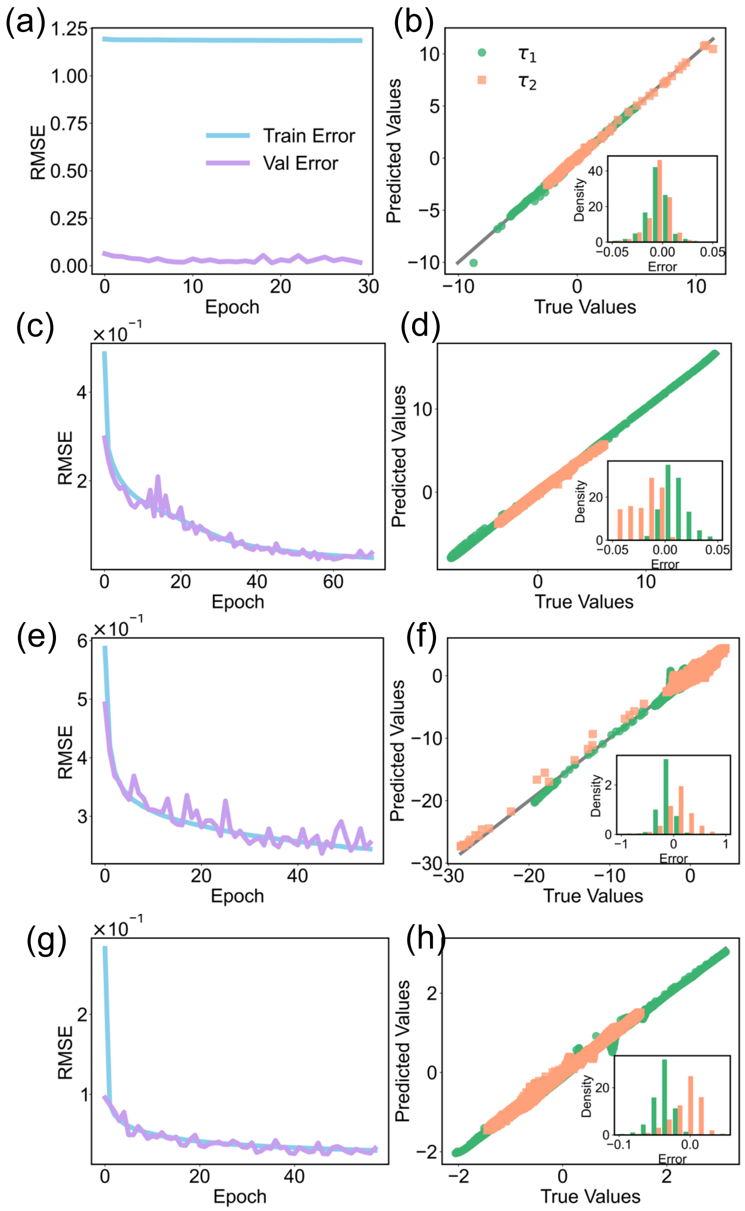

FIG. S13. Examples of successful tracking control using FNNs. The four rows show four examples of trajectories: chaotic Lorenz, circular, chaotic Mackey-Glass system with $\tau_{\rm mg} = 17$ and the eight-symbol. (a,c,e,g) Training RMSE of the loss function. (b,d,f,h) Predicted versus true values, with the insets displaying the statistics of the error between the predicted and true values. There exists a map between the state error terms and the control signal, and the FNN controller has the ability to output the proper control signal and track different trajectories.

desired observation state $\mathbf{y}_{\rm d}(t+dt)$, we obtain four inputs for the FNN: E_x, E_y, $E_{\dot{q}_1}$, and $E_{\dot{q}_2}$. At each step, we have two scalar outputs: the control signal $u = [\tau_1, \tau_2]$. Since the Cartesian positions of the end effector and the angular velocities have different magnitudes, it is useful to normalize the data for training, which will ensure that the FNN learns the signal in the input channels uniformly to avoid bias towards learning any particular input [20]. We use $z-$score

normalization [21]: $z = (x_z - \bar{x}_z)/S_z$ to normalize both the input and output, where $\bar{x}_z$ and S_z are the mean and standard deviation of the data x, respectively. We divide the data into a training and a testing segment, with 80% and 20% of the total data, respectively. In the training phase, we take 20% of the training data as the validation data for calculating the loss function. The training and testing results are shown in Fig. S13, where the four rows represent four examples of reference trajectories: chaotic Lorenz, circular, chaotic Mackey-Glass with $\tau_{\mathrm{mg}} = 17$, and the eight symbol. The first column records the loss function and the RMSE [22] versus the training epoch, which can be calculated by the sum of the absolute errors divided by the sample size. From the first column, it can be seen that the training and validation RMSEs decrease and then gradually approach a steady value. The second column of Fig. S13 shows the testing phase results: the normalized true values of the control signal $u = [\tau_1, \tau_2]$ and the predicted values as the output of the FNN, where the green circles and orange squares represent τ_1 and τ_2, respectively. The grey lines are for facilitating the interpretation of the results. Subtracting the predicted from the true values, we obtain the statistics of the errors, as shown in the four insets. These results indicate that, by designating the error terms as the input and the control signal as the output for the FNN, satisfactory learning and prediction can be achieved. That is, the FNN can successfully yield the mapping between the state errors and the control signal.

In general, the tracking and control of dynamic systems require a neural architecture that can maintain a memory of past inputs to process future inputs - time-series analysis. Recurrent neural networks (RNNs) have demonstrated success in such scenarios. However, RNNs and their advanced versions, such as LSTM and GRU, possess a high computational time complexity and may not be suitable for online applications. Reservoir computing (RC) overcomes the computational complexity by excluding the reservoir weights from training. Instead, the reservoir weights are fixed, and training is only necessary to tune the output matrix or the readout layer. which can be achieved quickly using linear regression.

FNNs are essentially RNNs unfolded in time and thus face the same challenges as RNNs. While FNNs may work well for the example of the two-link robotic arm with two degrees of freedom (2-DoF) demonstrated in this paper, RC is the preferred architecture for tracking higher degree-of-freedom dynamic systems operating at high speeds.

SUPPLEMENTARY REFERENCES

[1] Jaeger, H. The "echo state" approach to analysing and training recurrent neural networks-with an erratum note. *Bonn, Germany: German National Research Center for Information Technology GMD Technical Report* **148**, 13 (2001).

[2] Maass, W., Natschläger, T. & Markram, H. Real-time computing without stable states: A new framework for neural computation based on perturbations. *Neu. Comp.* **14**, 2531–2560 (2002).

[3] Appeltant, L. *et al.* Information processing using a single dynamical node as complex system. *Nat. Commun.* **2**, 1–6 (2011).

[4] Carroll, T. L. Optimizing memory in reservoir computers. *Chaos* **32**, 023123 (2022).

[5] Bishop, C. M. Training with noise is equivalent to tikhonov regularization. *Neural computation* **7**, 108–116 (1995).

[6] Surrogate optimization for global minimization of time-consuming objective functions - MATLAB. `https://www.mathworks.com/help/gads/surrogateopt.html`.

[7] Lorenz, E. N. Deterministic nonperiodic flow. *J. Atmos. Sci.* **20**, 130–141 (1963).

[8] Mackey, M. C. & Glass, L. Oscillation and chaos in physiological control systems. *Science* **197**, 287–289 (1977).

[9] Wernecke, H., Sándor, B. & Gros, C. Chaos in time delay systems: An educational review. *Phys. Rep.* **824**, 1–40 (2019).

[10] Rössler, O. E. Equation for continuous chaos. *Phys. Lett. A* **57**, 397–398 (1976).

[11] Zhao, H. *et al.* Connected fermat spirals for layered fabrication. *ACM Trans. Graph. (TOG)* **35**, 1–10 (2016).

[12] Sprott, J. C. Some simple chaotic flows. *Phys. Rev. E* **50**, R647 (1994).

[13] Lü, J. & Chen, G. Generating multiscroll chaotic attractors: theories, methods and applications. *Int. J. Bif. Chaos* **16**, 775–858 (2006).

[14] Lockwood, E. H. *A book of curves* (Cambridge University Press, 1967).

[15] Lorenz, E. N. Predictability: A problem partly solved. In *Proc. Seminar on predictability*, vol. 1 (Reading, 1996).

[16] Vlachas, P. R. *et al.* Backpropagation algorithms and reservoir computing in recurrent neural networks for the forecasting of complex spatiotemporal dynamics. *Neural Networks* **126**, 191–217 (2020).

[17] Kucuk, S. Optimal trajectory generation algorithm for serial and parallel manipulators. *Robot. Comp.-Inte. Manufact.* **48**, 219–232 (2017).

[18] Svozil, D., Kvasnicka, V. & Pospichal, J. Introduction to multi-layer feed-forward neural networks. *Chemome. Intell. Lab. Sys.* **39**, 43–62 (1997).

[19] Møller, M. F. A scaled conjugate gradient algorithm for fast supervised learning. *Neu. Net.* **6**, 525–533 (1993).

[20] Ormiston, R., Nguyen, T., Coughlin, M., Adhikari, R. X. & Katsavounidis, E. Noise reduction in gravitational-wave data via deep learning. *Phys. Rev. Res.* **2**, 033066 (2020).

[21] Jain, A., Nandakumar, K. & Ross, A. Score normalization in multimodal biometric systems. *Pat. Recog.* **38**, 2270–2285 (2005).

[22] Ji, L. & Peters, A. J. Assessing vegetation response to drought in the northern great plains using vegetation and drought indices. *Remote Sen. Environ.* **87**, 85–98 (2003).
